# Analysis of Specific Physical Fitness in High-Level Table Tennis Players—Sex Differences

**DOI:** 10.3390/ijerph19095119

**Published:** 2022-04-22

**Authors:** Francisco Pradas, Víctor Toro-Román, Ana de la Torre, Alejandro Moreno-Azze, Juan Francisco Gutiérrez-Betancur, Miguel Ángel Ortega-Zayas

**Affiliations:** 1 ENFYRED Research Group, Faculty of Health and Sports Sciences, University of Zaragoza, 22001 Huesca, Spain; franprad@unizar.es (F.P.); 678853@unizar.es (A.d.l.T.); amazze@unizar.es (A.M.-A.); maortega@unizar.es (M.Á.O.-Z.); 2 School of Sport Sciences, University of Extremadura, 10003 Cáceres, Spain; 3 Institute of Physical Education and Sport, University of Antioquia, Ciudadela de Robledo, Medellín 050036, Colombia; direducacionfisica@udea.edu.co

**Keywords:** table tennis, physical fitness, racket sport, exercise

## Abstract

Table tennis performance depends on multiple factors such as technique, tactics and fitness. Several studies have focused on investigating different technical-tactical variables. However, research analysing the specific physical qualities of this sport is scarce, particularly in the female sex. The aim of the present study was to assess the physical fitness variables associated with individual performance in elite table tennis players according to sex. Forty-eight elite players divided into males (*n* = 24; 25.38 ± 4.01 years) and females (*n* = 24; 22.33 ± 3.83 years) participated in the study. To determine physical fitness, participants performed vertical jump, hand grip strength, ergospirometry and lateral displacement tests (reaction time, displacement time and lateral acceleration). Male players showed higher values in vertical jump, hand grip strength and maximum oxygen consumption (*p* < 0.001). Likewise, male players moved laterally faster (*p* < 0.001). On the other hand, female players had a better reaction time towards the dominant side (*p* < 0.01). Elite male table tennis players showed better physical fitness compared to female players. Due to the scarcity of data on elite table tennis players, these results can serve as reference values for different table tennis practitioners.

## 1. Introduction

Table tennis is a very popular Olympic sport, played by more than 300 million people. Over the last decade, it has experienced great progress, with changes in its sport rules, such as the inclusion of plastic balls, increased ball diameter and weight, an 11-point scoring system and the introduction of time-outs [1]. This made the game more up-to-date and more engaging [2].

Table tennis is a racket sport characterized by high-speed and high-intensity playing actions dominated by intermittent physical efforts [3]. Metabolically, table tennis is classified as a mixed activity. Approximately 4% of the efforts generated during a match depend on anaerobic energy pathways corresponding to short duration and high intensity actions. Blood lactate concentrations during and after a match are known not to exceed 2 mmol-L^−1^ [1], which confirms the predominance of aerobic metabolism characteristic of the low intensity and recovery phases during the match, corresponding to 96% of the remaining energy metabolism [3,4]. However, alactic anaerobic endurance is very significant and important, even if it has a low percentage value, due to the fact that almost all the decisive actions of the game occur in efforts with an average duration of 3.5 s [1,5].

Table tennis is considered to be one of the fastest sports in terms of game speed [6]. Matches analysed in high-level competitions have produced situations in which the speed of the ball has reached 120 km-h^−1^, even exceeding 160 km-h^−1^ in certain game situations [7,8]. Due to the small size of the playing table and the high speed at which the game is played, a table tennis player has only a fraction of a second to simultaneously analyse the game, react, move and position properly to hit the ball in optimal conditions [9]. 

High-speed movements of the lower body, in which fast but short accelerations are combined with braking actions, coordinated with high-speed execution of different techniques performed by the dominant arm are some of the skills involved in table tennis [10]. These specific features of the game of table tennis require a specific physical profile, where skills such as speed, strength and endurance are of great importance, whilst not undervaluing other elements of great importance such as perceptual and decision motor processes [11].

Over the last decade, table tennis has become more physically demanding, due to the increasing intensity of the game and the pace of the actions [6]. Consequently, there is a growing interest in how far modern table tennis has altered the skills and fitness of athletes [12]. Indeed, athletes who aspire to reach the highest performance need to acquire excellent perceptual-motor and anticipation skills to achieve greater control of the game [6], as well as acquiring the skills to deal with the physical demands [6,13].

Whilst talent development in table tennis is a multidimensional process, elite table tennis demands significant perceptual-motor skills [14]. These table tennis skills are considered to be critical in the performance of technical skills and tactical situations [15]. In this sense, high levels of qualities such as speed, agility, coordination, reaction time, strength and flexibility are essential to perform the different techniques and tactics correctly [2,16,17,18].

Assessment of physical performance by means of test batteries enables monitoring of the athlete’s evolution in order to create individual training programs or to identify possible injuries [19]. The physical fitness of elite athletes has been investigated in different sports modalities [20,21,22]. Particularly, racket sports have shown an increase in research on this topic [19,23,24]. Specifically, Pradas et al. [19] showed elevated maximal oxygen uptake (VO_2max_) and vertical jump values in male padel players when compared to female padel players. In tennis players, Ulbricht et al. [25] reported that boys, in different categories, showed higher values of hand grip strength, sprinting and medicine ball throwing. However, in elite table tennis, fitness information is scarce [16,26], being non-existent in elite female players. Likewise, a large number of research studies on table tennis do not include female players as participants [3,4,27]. Due to the scarce research and the lack of data in this group, it seems necessary to carry out physical fitness research in female table tennis players with the aim of establishing preliminary reference data. Therefore, the aim of the present study was to evaluate the most relevant and specific fitness variables associated with individual performance of elite table tennis players according to sex.

## 2. Materials and Methods

### 2.1. Participants

Forty-eight elite table tennis players participated in the present study voluntarily. Participants were divided into 24 male players (25.38 ± 4.01 years; 69.96 ± 9.26 kg; 1.76 ± 0.06 m) and 24 female players (22.33 ± 3.83 years; 57.63 ± 6.19 kg; 1.66 ± 0.06 m). Written informed consent was obtained from the participants before beginning the investigation. The research protocol was reviewed and approved by the Clinical Research Ethics Committee of Aragon (Spain) (code: 19/2010) following the guidelines of the Ethical Declaration of Helsinki, updated at the World Medical Assembly in Fortaleza (2013) for research in humans. A code was assigned to each participant in order to maintain anonymity. Participants’ characteristics are shown in Table 1.

Participants were recruited from different clubs and had to comply with the following criteria: (i) be over 18 years of age; (ii) participate in the highest category of the Spanish league; (iii) play in international competitions (International Table Tennis Federation or European Table Tennis Union); (iv) no injury or illness during the investigation or at least 6 months prior to the study. 

### 2.2. Procedure

The study was carried out during the middle of the season. For a more objective assessment, not training the day before the assessments was required. Two days before the assessments, the players completed an ad hoc open-ended survey with additional information about their experience and the characteristics of their game. All assessments were carried out on the same day for each sex. Before the assessments, participants performed a 10-min warm-up based on general mobility and continuous running on the mat. All participants were familiarized with the different assessments. The fitness assessments were carried out in two different phases: in the first phase, anthropometric tests, accelerations, lateral displacements, reaction time, hand grip strength and vertical jump were performed. In the second phase, the maximal incremental test was performed.

### 2.3. Antrhopometric Measurement

Weight (kg) and height (m) data were collected using a balance (Seca 769, Seca, Hamburg, Germany) and measuring rod (Seca 220, Seca, Hamburg, Germany) with an accuracy of ±0.001 kg and 0.001 m when barefoot and as lightly clothed as possible. 

### 2.4. Vertical Jump Measurement

The squat jump (SJ) and the counter-movement jump (CMJ) test were established to assess vertical jumping due to their high reliability [28]. A jump mat system (Newtest Powertimer^®^, Oulu, Finland) was used to measure height and time flight during jumps. The guidelines proposed by Bosco et al. were followed during the test [29]. The watts were calculated indirectly using the formula of Sayers et al. [30]. Recovery was 30 s between jumps. The best jump of three attempts was selected for further analysis. 

### 2.5. Hand Grip Strength Assessment

Manual grip strength was measured with a Takei 5101 dynamometer (Takei Instruments Ltd., Tokyo, Japan). Participants performed two maximal voluntary contractions while fully extending the hands and arms. Dominant and non-dominant hands were assessed. The grip of the dynamometer was adapted to the hands of the participants [31]. The highest value of two attempts was selected for further analysis. Rest time between attempts was 2 min.

### 2.6. Accelerations, Lateral Displacements and Reaction Time Measurement

Players performed the Take-Off Reaction Test (Newtest Powertimer^®^, Oulu, Finland) in accordance with the protocol described by Castellar et al. [32]. Participants began the performance, left or right, from the table tennis base position after reacting to the red light (left or right) emitted randomly by an electronic device. Participants then left the contact mat and made a lateral run to the left or right photocells (placed 5 m from the mat). Participants completed 12 attempts (six to the left and six to the right) and the best result was recorded. All attempts were performed consecutively with a short rest period of 15–20 s between attempts. At the end of each attempt, participants returned to the mat, starting the next attempt when the base table tennis position was adopted.

The variables collected were: (a) reaction time: time elapsed between switching on the lights (right or left) and the moment when the participants took their foot off the mat; (b) acceleration: change in speed from the start of lateral displacement (right or left) until the photocell barrier was exceeded; (c) lateral displacement: time elapsed between switching on the lights (right or left) and the moment when the participants exceeded the photocell barrier. Anticipation was considered when players reached a reaction time of <150 ms. To avoid any possible impact of the upper limbs, the photocells were placed at the hips of the subjects. The lowest time of each assessment was considered the dominant side (DS) and the highest time the non-dominant side (NDS).

### 2.7. Maximal Incremental Test

A progressive maximal treadmill test (Pulsar HP, Cosmos, Nussdorf, Germany) was performed to determine different physiological parameters such as maximum heart rate (HRmax) and maximal oxygen uptake (VO_2max_). The test started at a speed of 8 km-h^−1^. During the test, 1 km-h^−1^ was increased every minute. The participants warmed up on the treadmill at a speed of 6 to 7 km-h^−1^ for 7 min before the test. The test was performed at a constant gradient of 1%. An Oxycon Pro gas analyser (Jaegger, Hanover, Germany) was used to determine VO_2max_. A heart rate monitor (Polar Team System, Kempele, Finland) was used to monitor heart rate.

### 2.8. Statistical Analysis

Data are presented as means ± standard deviation and range (min–max). The Shapiro–Wilks test was used to determine the normal distribution of the variables and the Levene test was used to determine the homogeneity of variances. Student’s *t*-test for unrelated samples was used to determine sex differences. A value of *p* ≤ 0.05 was considered statistically significant. Statistical analysis was performed with IBM^®^ SPSS^®^ Statistics version 22.0 (IBM Corp., Armonk, NY, USA). Effect size (ES) was calculated using the g of Hedge. ES of 0.2, 0.4, and 0.8 were considered small, moderate, and large, respectively [33].

## 3. Results

Table 2 shows the ergospirometric values obtained in the maximal incremental test. Significant differences in VO_2max_ were achieved in both, absolute values and relative to body mass (*p* < 0.001).

Table 3 shows the results obtained in hand grip strength. Male players had higher strength values in the dominant and non-dominant hand compared to female players (*p* < 0.001). 

Table 4 shows the results obtained in the vertical jump tests (SJ and CMJ). Male players obtained higher values in all the parameters evaluated compared to female players (*p* < 0.001).

Finally, Table 5 reflects the results obtained in lateral displacement, lateral acceleration and reaction time. Male players had a shorter time in lateral displacement and acceleration to both the DS and NDS (*p* < 0.001). However, female players had a shorter reaction time to the DS than male players (*p* = 0.007).

## 4. Discussion

The aim of the present study was to evaluate the most relevant and specific fitness variables associated with individual performance of elite table tennis players as a function of sex. Previous studies have analysed physical fitness in table tennis players [2,34,35]. However, previous studies have focused on youth players. The strength of the present research is the assessment of elite players’ fitness, especially female players, where literature is non-existent.

Maintaining high levels of hand grip strength is key to manipulating the racket and adapting to the repeated and continuous impacts of hitting the racket against the ball. Low rates of maximal isometric strength are related to the appearance of symptoms of neuromuscular fatigue that can have a negative influence on correct biomechanical execution, and therefore on technical expression [36]. Maintaining an optimal capacity for this quality can improve players’ performance, increasing the speed and power of each technical gesture [37]. Furthermore, it delays fatigue, avoiding unforced errors and maintaining an adequate technical and tactical performance throughout the match [38].

In the present study, male players showed higher levels of strength compared to female players in both hands (*p* < 0.001). The results of the present study regarding hand grip strength are lower than those described in other racket sports. Pradas et al. [19] observed mean hand grip strength values of 33.7 kg and 27.41 kg in the dominant and non-dominant hand, respectively, in Spanish elite female padel players. In men, mean values of 51.1 kg and 46.2 kg were obtained in the dominant and non-dominant hand respectively. In tennis players, Fernandez-Fernandez et al. [39] observed mean values of 49.8 kg in male and 35.7 kg in female elite U18 players. According to these findings, it seems evident that the forearm strength required for the execution of the different strokes in tennis or padel is greater than the one required in table tennis, which is associated with the different playing techniques [40,41]. These differences may be associated with a greater need for force levels due to the greater weight of the implement to be handled, as well as the mobile to be struck [38]. Maintaining high levels of maximal isometric strength may have a positive transfer with correct hitting technique, both in table tennis and in other racket sports. [42,43]. 

Although in table tennis the resistance that the upper body must overcome during the impact of the implement on the ball is relatively small, the force that must be applied to perform each type of technique during each stroke in each play, will be translated into translation and rotation speed of the ball. These two elements are very important in this sport because of their relevance to obtain high levels of performance, with an average of 4–5 strokes per play, sometimes exceeding 20 strokes [9], with a high number of these strokes being performed by topspin. This technique is performed at very high speed and with an important hitting power to cause a high rotation and speed on the ball [44]. The technical ability to significantly accelerate the racket is considered as a relevant factor affecting performance levels during play [45]. However, there are no studies that relate the levels of hand grip strength with the sporting success obtained, despite its relevance during the game. Maintaining optimal levels of hand grip strength could delay fatigue, avoiding unforced errors and maintaining an adequate technical performance throughout the match.

The maximum isometric strength obtained by the non-dominant arm is lower than that of the dominant arm in both sexes, similar to what has been found in other racket sports [19,46,47]. These differences are associated with the continued practice of this asymmetric sport from an early age [2]. Assessment of manual grip strength allows differentiation of upper limb asymmetries. Morphological asymmetry can impact sports performance as it can cause unfavorable functional changes, which in turn increases the risk of injuries and conditions caused by overexertion. Therefore, regular assessment is necessary in order to improve athletic performance and minimize the risk of injury [48].

Despite the low physical load that the racket–ball interaction in table tennis may involve in the different technical actions, an imbalance was found in the maximum isometric strength in the present study, with higher levels of strength in both sexes on the DS compared to the NDS. These results have also been observed in youth players [49] and elite [36]. Previously, Picabea et al. [26] described sex differences in Spanish table tennis players, although without distinguishing between the DS and NDS in a study with table tennis players. However, Martinez et al. [50] did take laterality into consideration in a study carried out on post-pubertal Spanish table tennis players, obtaining differences in both extremities when comparing both sexes, similar to what was found in this study. Pradas et al. [36] found that players with an offensive style of play have higher rates of maximal isometric strength in the upper limb, both dominant and non-dominant, than players with a defensive style of play. These differences appear to be greater in male players compared to female players [51]. It has been reported that the development of strength in these body segments can improve players’ performance, increasing the speed and power of each technical skill [37] as occurs when an offensive style of play is developed. Nevertheless, in this study it has not been possible to perform this type of analysis due to the lack of statistical power, as there are very few players with a defensive style of play. Discrepancies between sexes in the results could be related to physiological differences such as a larger development of testosterone in men, which is related to strength and muscle mass [52]. 

VO_2max_ is considered to be an optimal marker of a player’s aerobic fitness [13]. In this regard, studies of elite Asian and European players have revealed VO_2max_ ranges between 43.9 and 67.9 mL.kg.min^−1^ [53,54], which are values found to be within the range of those reported in the present study for men. In comparison with other racket sports, a lower VO_2max_ value was found among the participants in the present study compared to padel players, both in male and female players. However, in tennis players, mean values of 58 mL.kg.min^−1^ were observed after a specific test, with higher values than in the present study. Moreover, mean values of 46 mL.kg.min^−1^ have been observed in badminton players and 36.4 mL.kg.min^−1^ in female players, being lower than the results obtained in the present study. Like other sports, table tennis has an aerobic component. High levels of endurance allow one to maintain strokes for a longer time in a game and to recover more quickly during a game [13]. It is therefore important to maintain high levels of maximum oxygen consumption, hence the importance of its training and assessment.

The assessment of the behaviour of lower body strength in its active (impulsive) and reactive (elastic-impulsive) manifestations is of great interest in this sport. The assessment of these strength performances was carried out by means of the SJ and CMJ tests [29,55]. Differences between male and female values obtained were shown (*p* < 0.001), with results in both performance parameters higher in men, similar to what has been found in different studies [56,57]. Picabea et al. [26] found results in Spanish table tennis players slightly lower for CMJ in males and slightly higher values in females. Although, these results cannot be considered because the Abalakov test was used. More recently, Picabea et al. [16] obtained values similar to the present study for CMJ height in senior Spanish table tennis players.

If the results of this research are compared with other racket sports, it can be seen that table tennis has better values than high-level padel, especially for SJ in the female sample [19,38,58]. However, in male tennis players, higher values were reported for the CMJ than in table tennis [39], and similarly in badminton [59]. The differences observed in the present study between sexes can be explained by a better neuromuscular and coordination capacity of the lower body in male [52]. Likewise, similar to what is indicated for maximal isometric grip strength, players with an offensive style of play obtain better results than those with a defensive style of play [56]. Offensive play is characterised by speed and explosiveness, probably due to the increased development of players’ muscle mass, as a direct relationship between muscle cross-sectional area and strength indices [52]. In addition, the significantly higher height/impulsive values obtained by players in the vertical jump tests could be due to anthropometric and strength differences between both sexes, a circumstance that is also present in various sports disciplines [58].

The relationship between ocular stimulus and the elapsed time for a movement to occur in a given body segment, either upper or lower limb, has been previously investigated in the sport of table tennis [32,60,61]. Indeed, this is due to its importance in establishing a quality motor programme of response to the speed of ball displacement during play [32]. In this sense, the capacity for a fast reaction of the player to a visual stimulus is key to achieve an optimal performance in this sport [62,63]. Due to the small size of the game table and the high speed at which the game is played, it is considered one of the fastest sporting disciplines in existence [64].

Findings obtained in this research show that male players have better lateral displacement speed and acceleration to both sides, while female players react faster than male players to both sides, coinciding with previous findings in this sport [32]. The difference in reaction time could be due to anthropometric differences between sexes. Female players might have an advantage over male players reaction time because they are generally shorter in height compared to men. Therefore, the neural impulses involved in the production of a motor response have less distance in women than in men [65]. Reaction time is considered to be one of the most important features of table tennis performance [32]. Despite the scarcity of existing studies analysing these variables in racket sports [62], evidence of this is that table tennis players, both male and female, perform better than elite padel players [66], showing an even higher dominance of perceptual-motor skills than table tennis players, probably related to the speed at which this sport is performed [67].

This research has some limitations: (i) specific hitting tests were not carried out; (ii) no distinction was made by age, which could affect the average results, especially in males whose age range was wider; (iii) only the lower limbs were taken into consideration in the lateral displacement test, ignoring the movements of the upper limbs; (iv) the style of play (offensive, defensive or mixed) was not taken into consideration, nor the materials used in the racket that indicate the type of game to be developed in this sport. Future studies could investigate the relationship between playing style and physical fitness.

## 5. Conclusions

Male elite table tennis players showed better fitness values compared to elite female players. Specifically, male players obtained better results in hand grip strength, VO_2max_, vertical jump (SJ and CMJ), displacement and lateral acceleration. Nevertheless, female players showed a better reaction time. 

Knowledge of the physical abilities of table tennis players can help coaches to create specific training programmes for optimal physical preparation as well as to select potential talents. Due to the scarcity of data regarding physical fitness in elite table tennis players, these results can serve as initial reference values for different table tennis practitioners.

## Figures and Tables

**Table 1:** Descriptive characteristics of the participants (*n* = 48).

		Male (*n* = 24)	Range	Female (*n* = 24)	Range
Age (years)		25.38 ± 4.01	19–38	22.33 ± 3.83	10–22
Weight (kg)		69.96 ± 9.26	50.80–89.60	57.63 ± 6.19	48.30–69.80
Height (m)		1.76 ± 0.06	1.62–1.88	1.66 ± 0.06	1.52–1.75
BMI (kg-m^−2^)		22.60 ± 2.34	18.86–27.25	20.94 ± 1.62	18.33–24.42
Experience (years)		16.04 ± 4.12	9–30	13.25 ± 3.86	10–22
Laterality (%)	Left-handed	29.2	-	16.6	-
	Right-handed	70.8	-	83.3	-
Playing style (%)	Offensive	95.9	-	87.5	-
	Defensive	4.1	-	12.5	-

BMI: body mass index.

**Table 2:** Ergospirometric values obtained in the maximum incremental test.

	Male (*n* = 24)	Range	Female (*n* = 24)	Range	*p*	ES
VO_2max_ (l-min^−1^)	3.72 ± 0.73	2.65–5.25	2.54 ± 0.39	1.72–3.22	<0.001	1.16
VO_2max_ (mL-kg-min^−1^)	53.00 ± 6.03	41.00–63.10	44.21 ± 5.69	32.65–54.30	<0.001	1.52
HR_max_ (beats-min^−1^)	194.63 ± 6.38	176.0–207.0	195.54 ± 4.69	184.0–200.0	0.904	0.17

VO_2max_: maximal oxygen uptake; HR_max_: maximum heart rate; ES: effect size.

**Table 3:** Maximum hand grip force.

	Male (*n* = 24)	Range	Female (*n* = 24)	Range	*p*	ES
Dominant (kg)	44.48 ± 6.15	35.20–55.00	28.89 ± 3.52	23.50–37.60	<0.001	2.15
Non dominant (kg)	41.04 ± 6.85	28.50–54.10	24.61 ± 3.60	20.50–33.00	<0.001	2.90

ES: effect size.

**Table 4:** Vertical jump test results.

	Male (*n* = 24)	Range	Female (*n* = 24)	Range	*p*	ES
Flight time SJ (s)	0.515 ± 0.04	0.430–0.573	0.434 ± 0.04	0.332–0.515	<0.001	1.62
Height SJ (cm)	32.83 ± 5.33	22.60–40.30	23.67 ± 5.00	13.50–32.50	<0.001	1.72
Power SJ (W)	3106.78 ± 544.88	1867.2–4141.7	1991.98 ± 470.69	984.1–2898.5	<0.001	1.85
Power SJ (W-kg^−1^)	44.32 ± 4.89	33.17–51.37	34.28 ± 5.88	20.08–44.05	<0.001	1.67
Flight time CMJ (s)	0.539 ± 0.04	0.463–0.609	0.467 ± 0.05	0.390–0.567	<0.001	1.54
Height CMJ (cm)	35.74 ± 5.65	26.30–44.40	27.17 ± 6.13	18.60–39.20	<0.001	1.48
Power CMJ (W)	3269.16 ± 550.20	2016.7–4258.4	2220.81 ± 482.56	1421.9–3151.7	<0.001	1.33
Power CMJ (W-kg^−1^)	46.63 ± 4.47	38.42–53.51	38.30 ± 6.00	29.02–49.36	<0.001	1.71

CMJ: counter movement jump; SJ: squat jump, ES: effect size.

**Table 5:** Lateral displacement time, lateral acceleration and reaction time according to sex.

	Male (*n* = 24)	Range	Female (*n* = 24)	Range	*p*	ES
Displacement time NDS (s)	1.941 ± 0.104	1.77–2.15	2.208 ± 0.105	1.92–2.43	<0.001	0.78
Displacement time DS (s)	1.913 ± 0.134	1.63–2.16	2.178 ± 0.007	2.05–2.32	<0.001	0.84
Reaction time NDS (s)	0.692 ± 0.108	0.50–0.94	0.636 ± 0.139	0.49–1.10	0.218	0.11
Reaction time DS (s)	0.724 ± 0.143	0.49–0.89	0.581 ± 0.139	0.13–0.72	0.007	0.30
Acceleration NDS (m-s^2^)	1.249 ± 0.149	0.83–1.43	1.572 ± 0.104	1.32–1.69	<0.001	0.68
Acceleration DS (m-s^2^)	1.189 ± 0.196	0.91–1.58	1.597 ± 0.126	1.37–1.92	<0.001	0.85

DS: dominant side; NDS: non-dominant side; ES: effect size.

## Data Availability

Not applicable.
